# Acquired pure red cell aplasia: updated review of treatment

**DOI:** 10.1111/j.1365-2141.2008.07216.x

**Published:** 2008-05-28

**Authors:** Kenichi Sawada, Naohito Fujishima, Makoto Hirokawa

**Affiliations:** 1 Department of Internal Medicine III, Division of Haematology, Akita University Graduate School of MedicineAkita, Japan; 2 Oncology Centre, Akita University Graduate School of HospitalAkita, Japan

**Keywords:** pure red cell aplasia, corticosteroids, cyclosporine A, cyclophosphamide, alemtuzumab, rituximab

## Abstract

Pure red cell aplasia (PRCA) is a syndrome characterized by a severe normocytic anaemia, reticulocytopenia, and absence of erythroblasts from an otherwise normal bone marrow. Primary PRCA, or secondary PRCA which has not responded to treatment of the underlying disease, is treated as an immunologically-mediated disease. Although vigorous immunosuppressive treatments induce and maintain remissions in a majority of patients, they carry an increased risk of serious complications. Corticosteroids were used in the treatment of PRCA and this has been considered the treatment of first choice although relapse is not uncommon. Cyclosporine A (CsA) has become established as one of the leading drugs for treatment of PRCA. However, common concerns have been the number of patients treated with CsA who achieve sustained remissions and the number that relapse. This article reviews the current status of CsA therapy and compares it to other treatments for diverse PRCAs.

Pure red cell aplasia (PRCA), a disorder first characterized in 1922 (Kaznelson, 1922), is a syndrome characterized by severe normochromic, normocytic anaemia associated with reticulocytopenia and absence of erythroblasts from an otherwise normal bone marrow. PRCA may appear as a congenital disorder or occur as an acquired syndrome. The acquired form of PRCA presents either as an acute self-limited disease, predominantly seen in children, or as a chronic illness that is more frequently seen in adults. It may present as a primary haematological disorder in the absence of any other disease, or secondary to parvovirus infection, collagen vascular disease, leukaemia, lymphoma, thymoma, solid tumors, treatment with recombinant human erythropoietin (EPO) or other drugs, ABO-incompatible haematopoietic stem cell transplantation and pregnancy. Depending on the cause, the course can be acute and self-limiting or chronic with rare spontaneous remissions (Dessypris, 1988; Dessypris & Lipton, 2004).

Primary, or secondary PRCA not responding to treatment of the underlying diseases, is treated as an immunologically-mediated disease, based on a number of studies implicating a pathological role of serum auto-antibodies, natural killer (NK) cell-mediated or T lymphocyte-mediated effects impairing various stages and mechanisms of erythropoiesis as extensively reviewed by Fisch *et al* (2000). The major objective in the treatment of PRCA is to induce a remission with the recovery of erythropoiesis, thus providing relief from transfusions and avoiding transfusion-associated problems. The therapeutic plan usually focuses on the sequential use of various immunosuppressive therapies until a remission is obtained. Remissions have been achieved by treatment with corticosteroids (CS), cyclophosphamide (CY), cyclosporine A (CsA), anti-thymocyte globulin (ATG), splenectomy, and plasmapheresis (Dessypris & Lipton, 2004). More recently, the efficacies of the anti-CD20 monoclonal antibody, rituximab (Zecca *et al*, 2001), and anti-CD52 monoclonal antibody, alemtuzumab (Willis *et al*, 2001), to induce remissions of therapy-resistant PRCA have also been reported.

In general, remission can be easily achieved in the majority of patients. To date, the efficacy of CS, CY and CsA for patients with primary or secondary PRCA has been reported to be between 30–62%, 7–20% and 65–87%, respectively (Clark *et al*, 1984; Dessypris, 1988; Raghavachar, 1990; Marmont, 1991; Lacy *et al*, 1996; Mamiya *et al*, 1997). The efficacy of a combination of CY and CS for refractory patients has been reported to be between 40–60% (Clark *et al*, 1984; Dessypris, 1988, Mamiya *et al*, 1997). Since the initial cases were successfully treated by Totterman *et al* (1984), CsA has established itself as one of the leading drugs for the treatment of PRCA. However, concern has centred around the precise number of patients treated with CsA who achieve a sustained remission and the number who relapse. In 1988, Dessypris pointed out that treatment of PRCA with CsA appeared to be very promising, but that such treatment should be considered still experimental, and that further studies were necessary to determine the effectiveness of this drug, the optimal and least toxic dosage, the minimum duration of therapy for induction of remission, and whether or not there was a need for maintenance treatment (Dessypris, 1988). An advantage of CsA therapy for PRCA has long remained unclear, as comparing one therapeutic approach to another has been almost impossible because the disease is so rare that controlled studies could not be performed. However, the number of patients treated with CsA has accumulated over two decades, which made it possible to conduct an analytical study. The present paper reviews the current status of CsA therapy, comparing it to other treatments for the diverse types of acquired PRCA except for transient erythroblastopenia of childhood.

## Diagnosis and initial evaluation

Pure red cell aplasia in adults can be easily diagnosed when isolated anaemia, in the presence of normal white cell and platelet counts, is associated with a marrow of normal cellularity in which there is an almost complete absence of erythroblasts but normal myeloid cells and megakaryocytes (Dessypris & Lipton, 2004). The classification of the clinical course (acute or chronic) and pathogenesis, such as secondary or idiopathic (no definite underlying disease) is essential to select the optimal therapeutic modality. Evaluations for the possible causes of PRCA should include a previous history of drug use and toxins or infections, liver and kidney functions, immunological examination including auto-antibodies, a bone marrow examination including morphology, chromosome and rearrangement of T cell receptor (TCR) analysis, peripheral-blood flow cytometry, virological examination including parvovirus B19 DNA, and computed tomography and/or magnetic resonance imaging examinations to rule out the presence of thymoma and neoplasms.

Today, a careful assessment of the increase of large granular lymphocytes (LGLs) is especially critical and an analysis of immunophenotype and TCR rearrangement of lymphocytes may be essential for ruling out LGL leukaemia, also referred to as granular lymphocyte proliferative disorders (GLPD) (Oshimi *et al*, 1993) or lymphoproliferative disease of granular lymphocytes (LDGL) (Go *et al*, 2001). LGL leukaemia was the most common underlying disease of secondary PRCA in a single institutional study from the United States, and the second most common cause in Japan (Lacy *et al*, 1996; Mamiya *et al*, 1997; Sawada *et al*, 2007). Since the diagnosis of LGL leukemia is somewhat difficult in patients without lymphocytosis, this group of patients can be misdiagnosed as idiopathic PRCA although LGL leukemia-associated PRCA may require a different treatment for the primary disease. It is a heterogeneous disorder characterized by a persistent increase in the number of peripheral blood LGLs, and the majority of patients have a clonal rearrangement of T-cell receptors (Oshimi *et al*, 1993; Semenzato *et al*, 1997; Chan *et al*, 2001). Clonal disorders of LGLs arise from either mature T lymphocytes or NK cells, and may be indolent or behave as an aggressive disease. T-cell LGL leukaemia is the most common form of clonal LGL disorders and most cases behave in an indolent fashion. Neutropenia is the most frequent cytopenia in T-cell LGL leukaemia, and anaemia occurs in 48% of the patients (Loughran, 1993; Lamy & Loughran, 1998, 2003).

The evidence of a granular lymphocytosis greater than 2 × 10^9^/l lasting for more than 6 months has been regarded as the criteria for defining the disease (Loughran & Starkebaum, 1987; Semenzato *et al*, 1987; Oshimi, 1988). However, the normal range for peripheral blood LGL counts is 0·223 ± 0·099 × 10^9^/l (Loughran *et al*, 1987) and clonal disease has been documented in 8% of patients when absolute LGL counts are between 0·6 to 1·0 × 10^9^/l (Loughran, 1993). Thus, an expansion of a restricted LGL subset demonstrates the diagnosis of LGL-leukaemia and a 6-month follow-up criterion is not necessary when clonality is established (Semenzato *et al*, 1997). The characteristic finding is the presence of increased numbers of LGL, usually identified by a greater size than normal lymphocytes, abundant pale cytoplasm, and prominent azurophilic granules. However, these features may vary, even among cells from the same patient (Loughran, 1993). The granulation can range from fine to coarse, and some cells may have otherwise characteristic features but lack granules (sometimes called large agranular lymphocytes) (Bassan *et al*, 1986). Occasionally, clonally expanded lymphocytes with a characteristic CD3^+^, CD57^+^ phenotype may not have LGL morphology on a peripheral smear (Ahern *et al*, 1990) but may represent *in vivo* antigen-activated cytotoxic effector T cells. An increase of CD3^+^/CD56^−^ or CD3^−^/CD56^+^ cells by peripheral-blood flow cytometry and/or an inverted CD4^+^/CD8^+^ cell ratio (<1·0) suggests the existence of LGL leukaemia (Gonzales-Chambers *et al*, 1992).

## Initial management

During the initial evaluation, red cell transfusions can be given as necessary. In cases supposed to be primary idiopathic PRCA, it would be preferable to wait for at least a month before instituting specific treatment, with the rationale that 10–12% of PRCA patients run a short and self-limited course (Dessypris, 1988). If, after such a waiting period, no signs of recovery of erythropoiesis appear, specific treatment should be instituted. Many of the secondary PRCAs are due to drugs and disappear when the drug is stopped. Those secondary to parvovirus B19 can be treated with intravenous immunoglobulin. Secondary PRCA not responding to treatment of the underlying diseases and primary PRCA are treated as immunologically-mediated diseases.

## Immunosuppressive therapy

### Corticosteroids

Corticosteroids (CS) were the first immunosuppressive drugs used in the treatment of PRCA and so far have been considered the treatment of first choice, especially in young adults (Clark *et al*, 1984; Dessypris, 1988; Charles *et al*, 1996; Dessypris & Lipton, 2004). The details of CS therapy are described elsewhere (Dessypris, 1988; Dessypris & Lipton, 2004). In an era when CsA was not yet available, Clark *et al* (1984) reported the largest series of PRCA patients receiving immunosuppressive therapy and showed that 10/27 (37%) patients with acquired PRCA responded to CS within a mean period of 2·5 weeks. Comparable results of CS treatment were obtained at other institutions, ranging from 30–62% (Dessypris, 1988; Raghavachar, 1990; Marmont, 1991; Lacy *et al*, 1996; Mamiya *et al*, 1997). One of the important drawbacks of CS is that relapse is not uncommon: 80% of patients relapsed, as the dosage was tapered, during the 24 months after remission (Clark *et al*, 1984). The principal reason for discontinuing the drug, despite subsequent recurrence of anaemia, was the presence of unacceptable side effects, such as myopathy, infection, hyperglycemia, and compression fractures at the dose required to maintain remission. Treatment of relapses was successful, with 10/13 (77%) patients entering a second or third remission, and the median survival in patients with primary idiopathic PRCA was 14 years (Clark *et al*, 1984). Cytotoxic drugs administered in combination with CS were the most effective form of treatment in this study, producing 18/32 (56%) remissions. Although such vigorous immunosuppressive treatment is capable of inducing and maintaining remission in a majority of patients, it carries increased risks of serious infections, malignancy, and sterility (Clark *et al*, 1984). Thus, an individualized approach to management of PRCA has been widely accepted, i.e. escalating therapy in proportion to the severity of the disease for those patients who have failed CS therapy.

### Cyclosporin A

Raghavachar (1990) reviewed the treatment of PRCA, focusing on the results of cyclosporin A (CsA) therapy, in 43 patients. He showed that the overall response rate to CsA is excellent (65%) and proposed that CsA should be the first drug to be given in acquired PRCA. Of note is that a high dosage was used in order to obtain these results (12 mg/kg per day). Comparable results of CsA treatment have been obtained at other institutions, ranging from 65–87% (Dessypris, 1988; Marmont, 1991; Means *et al*, 1991; Lacy *et al*, 1996; Mamiya *et al*, 1997; Sawada *et al*, 2007). Mamiya *et al* (1997) reviewed the clinical features of 150 patients with acquired PRCA in Japan. In their surveillance, CsA was given to 38 patients in a daily dose of 200–600 mg (most often 200–300 mg) and 31 (82%) showed haematological recovery. The response rate to CsA was 87% in the patients with primary PRCA and 73% in those with secondary PRCA, which encouraged them to recommend CsA therapy as first-line therapy for this disease.

Recently, The Japan PRCA Collaborative Study Group conducted a nationwide survey in Japan between 1990 and 2006 (Sawada *et al*, 2007). From a total of 185 patients, consisting of 73 primary idiopathic and 112 secondary PRCA cases, 62 patients with primary idiopathic PRCA were evaluated, which is the largest and the longest follow-up study so far. Although a retrospective one, this study, for the first time, answered many of the unknown questions concerning CsA therapy. The remission induction therapies for these patients by CsA and CS produced remissions in 74% and 60% of patients, respectively. The initial dose of CsA for the responding patients was 4·8 ± 1·2 mg/kg (mean ± SD, *n* = 23) with a range of 2·9–7·6 mg/kg body weight. Patients treated with CsA alone (*n* = 23) became transfusion-independent by 82 ± 200 d, with a range up to 910 d, after the start of therapy. Fifteen patients (65%) achieved transfusion-independence within 2 weeks, 17 patients (74%) within 1 month and 18 patients (78%) within 3 months. Salvage immunosuppressive treatment achieved remissions in 58 patients (94%). Forty-one and 15 patients were maintained on CsA ± CS (CsA-containing group) or CS alone (CS-group), respectively. The median relapse-free survival (RFS; estimated as transfusion-free survival) in the CsA-containing group was 103 months, significantly longer (*P* < 0·01) than that seen in the CS-group (33 months). Thus, combined CsA therapy can sustain a longer duration of initial remission than CS, however, discontinuation of maintenance therapy was strongly correlated with relapse (*P* < 0·001) and caused relapses with a median of 3 months with a range of 1·5–40 months. In contrast, 88% of relapses in the CS-group occurred during maintenance prednisolone (PSL) therapy (Sawada *et al*, 2007).

Tötterman *et al* (1989) also reported that PRCA patients did not remain in remission after CsA was stopped. An important question is whether or not the maintenance of patients in remission may have a beneficial influence on survival. One study (Sawada *et al*, 2007) reported an estimated median OS of the CsA-containing group of 12 years, which was not significantly different than the CS-group, while the 10-year OS in all patients was 95% and the median OS had not yet been reached in all patients. Of importance is the fact that the CsA is required to maintain remissions (Sawada *et al*, 2007) and the decreased probability of relapse and requirement of red cell transfusions reduces the dangers of hemolysis, infections and iron overload with possible superoxide damage to body tissues. CsA is more expensive than CS and requires renal function to be monitored, but it seems to be important to prevent relapses and to sustain remissions in primary idiopathic PRCA. Although higher doses of CsA, such as 12 mg/kg per day have been used for patients with PRCA (Raghavachar, 1990), this dosage has been toxic for Japanese PRCA patients. Since cytochrome P-450 isoenzymes, involved in CsA metabolism, have a variable frequency of a reduced function allele depending on race and each individual (Bladford, 2004), the most important therapeutic index should be trough CsA levels. Caucasian patients with anti-EPO antibody-related PRCA have been successfully treated with CsA alone at a dose of 200 mg/d (or 100 mg twice daily) (Verhelst *et al*, 2004; Rossert *et al*, 2005). Since organ transplantations have shown that long-term immunosuppression is associated with post-transplant malignancies (Cattran *et al*, 1995; Young *et al*, 2006), continuous and careful follow-up is required for patients receiving long-term CsA therapy.

### Cytotoxic immunosuppressive drugs

Patients with an absolute contraindication for CsA or patients refractory to CsA may be treated with CS or a combination of CS and other immunosuppressives. Cyclophosphamide (CY) has been the principal alkylating agent utilized as an immunosuppressive drug in PRCA. The details of CY therapy are described elsewhere (Dessypris, 1988; Dessypris & Lipton, 2004). The initial dosage of CY is 50 mg/d p.o. and PSL at a dose of 20–30 mg/d is added in the absence of any contraindication. If the white blood cell and platelet counts allow, it is increased by 50 mg weekly or biweekly to a maximum of 150 mg daily until remission occurs or bone marrow suppression develops. The mean time to response is approximately 11 to 12 weeks with an overall response rate of 40 to 60%. When response occurs, the dose of PSL is tapered, and then the dose of CY is progressively decreased and eventually discontinued after 3–4 months from the time of normalization of haematocrit.

The duration of remission induced by CY seems to be prolonged as compared to remissions induced by CS (Clark *et al*, 1984; Firkin & Maher, 1988; Go *et al*, 2001). LGL leukaemia-associated PRCA has been primarily treated with chemotherapy, such as CY with or without CS, CsA, CS or methotrexate (Dhodapkar *et al*, 1994; Loughran *et al*, 1994; Lacy *et al*, 1996; Yamada *et al*, 1997; Sood *et al*, 1998; Hamidou *et al*, 2000; Go *et al*, 2001; Battiwalla *et al*, 2003; Osuji *et al*, 2006). The combination of CY plus CS is associated with a longer duration of response than CS alone (Dhodapkar *et al*, 1994; Lacy *et al*, 1996; Go *et al*, 2001). The overall response to initial CY ± CS therapy has been reported to be 66 to 100 % (Yamada *et al*, 1997; Go *et al*, 2003) and the median duration of response is 32 months (Go *et al*, 2003).

In one study, none of the patients with a response to cytotoxic agents had relapses (Lacy *et al*, 1996), but the other studies reported that a substantial number of patients relapsed when the CY was withdrawn (Zaentz *et al*, 1976; Clark *et al*, 1984). Maintenance CY therapy may prevent relapse, however, recognition of a variety of toxicities, particularly concerns about the long-term risk of malignancy and gonadal toxicity, often lead clinicians to consider less toxic alternative medications whenever possible (Csuka *et al*, 1986; Hoffman *et al*, 1992). These toxicity risks from alkylating agents are related to the cumulative dose of the medication (Reinhold-Keller *et al*, 2000) and the duration of therapy (Radis *et al*, 1995). Thus, the best role of CY therapy for PRCA might be to induce remissions using oral treatment lasting not longer than six months, with a switch to less toxic medication, such as CsA, for maintenance, but no controlled studies exist and this is purely speculative. It has been reported that crossover to azathioprine was effective in patients initially unresponsive to CY and vice versa (Firkin & Maher, 1988).

### Anti-thymocyte globulin

In the largest series of nine PRCA patients treated with anti-thymocyte globulin (ATG) at a dose of 15 mg/kg per day for 10 d, six responded to therapy, five with normal haematocrits and one with a stable haematocrit of 32% (Abkowitz *et al*, 1986). Three remained in complete remission and two relapsed, which suggests that ATG is an effective form of treatment. However, this is an expensive and confining therapy that requires hospitalization because of a possible anaphylactic reaction.

### Alemtuzumab

The anti-CD52 monoclonal antibody alemtuzumab (Campath-1H) is a humanized IgG_1κ_ monoclonal antibody directed against the CD52 antigen, present on B and T lymphocytes, NK cells, monocytes/macrophages, dendritic cells and eosinophils but not on human haemopoietic stem cells (Hale, 2001). The rationale behind the use of alemtuzumab in refractory cytopenias is that T-lymphocytes are thought to play an important role in the pathogenesis of autoimmune cytopenias, as they are involved in the control of expansion of immunoglobulin-producing, auto-reactive B-lymphocyte clones (Willis *et al*, 2001). Willis *et al* (2001) reported the effect of treatment with alemtuzumab followed by CsA administration in 21 patients with severe and life-threatening autoimmune cytopenias including four patients with PRCA. A response was seen in 2/4 patients with PRCA, although one patient relapsed at 7 months when the CsA blood level was suboptimal. One patient with PRCA in association with low-grade non-Hodgkin lymphoma died from high-grade transformation at 16 months while still in remission from the PRCA. Ru and Liebman (2003) reported two patients with chronic lymphocytic leukaemia (CLL) and a CD8 T-LGL leukaemia who were refractory to multiple treatments for PRCA. When both patients were treated with alemtuzumab, there was a rapid increase in the reticulocyte count that occurred as early as the third infusion. At the time reporting, both patients had been in PRCA remission for 9 and 5 months, respectively. These results indicated that alemtuzumab is an alternative option in the treatment of patients with refractory PRCA, however, relapse can occur and a significant number of patients need maintenance therapy with CsA after alemtuzumab treatment. Therefore, it remains uncertain whether alemtuzumab can induce a maintenance-free haematological response in PRCA. The use of alemutuzumab should be limited to patients with PRCA who are refractory to conventional immunosuppressive therapy because of the limited information and high risk of infections.

### Rituximab

Rituximab is a genetically engineered chimeric mouse/human monoclonal antibody that targets the CD20 molecule present on mature B cells, which are the precursors of autoantibody-producing plasma cells. Rituximab can selectively deplete B-cells by mechanisms including antibody-dependent cell-mediated cytotoxicity, complement-mediated cytotoxicity and inhibition of cell proliferation with direct induction of B-cell apoptosis (Smith, 2003). Rituximab has been found to be useful in treating primary autoimmune hemolytic anaemia and thrombocytopenia (Quartier *et al*, 2001; Stasi *et al*, 2001) and several reports have shown that PRCA was successfully treated with rituximab in patients with B-cell lymphoproliferative disorders mainly consisting of CLL (Batlle *et al*, 2002; Ghazal, 2002; Gupta *et al*, 2002; Hegde *et al*, 2002; Ru & Liebman, 2003; Pantelidou *et al*, 2004; Narra *et al*, 2006). The maximum time for onset of response was 4 weeks. In two cases, where PRCA and the primary disorder were simultaneously diagnosed, rituximab led to response in both PRCA and the primary disorder (Narra *et al*, 2006). One patient had a response to rituximab lasting only 4 weeks, but then responded to alemtuzumab (Ru & Liebman, 2003). No significant side effects were reported in any of these patients treated with rituximab. Dungarwalla *et al* (2007) have shown that the results of rituximab therapy in patients with severe, resistant and life-threatening PRCA refractory to conventional immunosuppression are disappointing. In their pilot study, all three of these patients with idiopathic PRCA did not respond to a conventional dose of rituximab (375 mg/m^2^) weekly for 4 weeks.

## Other therapeutic options

### Intravenous immunoglobulin

In immunocompromised hosts, such as recipients of organ transplantation (Wong *et al*, 1999), patients infected with human immunodeficiency virus (HIV) (Frickhofen *et al*, 1990) or receiving chemotherapy (Song *et al*, 2002; Isobe *et al*, 2004), acute or chronic anaemia can develop following parvovirus B19 infection due to the lack of the production of specific antibodies. Chronic B19 infection-related PRCA is a treatable anaemia and demonstration of the virus DNA in blood by the polymerase chain reaction or dot-blot hybridization assays is essential. Intravenous immunoglobulin (IVIG) contains neutralizing antibody against parvovirus B19 and has been reported to be effective for chronic B19 infection-related anaemia in immunocompromised hosts. Recurrence of anaemia is common in HIV-infected patients with low CD4^+^ T-cell counts (<0·08 ∼ 0·1 × 10^9^/l) and requires additional IVIG (Ramratnam *et al*, 1995). Anti-retroviral therapy may resolve chronic anaemia in HIV-infected patients (Mylonakis *et al*, 1999). IVIG may also be effective for PRCA due to parvovirus B19 in patients treated with rituximab (Sharma *et al*, 2000; Song *et al*, 2002). Alemtuzumab causes prolonged, severe CD4 and CD8 lymphopenia (Keating *et al*, 2002) and PRCA due to parvovirus B19 infection in a patient with cutaneous T-cell lymphoma treated with alemtuzumab has been reported (Herbert *et al*, 2003). Tacrolimus (FK506) is often associated with chronic B19 infection-associated anaemia in organ transplant recipients and cessation of tacrolimus or replacement with other immunosuppressants results in an improvement of anaemia (Wong *et al*, 1999).

### Thymectomy

Surgical resection of thymomas has been recommended as the initial treatment of thymoma-associated PRCA, with an expected haematological response rate of 25–30% (Zeok *et al*, 1979). In recent reports, resection of the thymoma by itself was effective in remitting the anaemia in only a small percentage of patients (Mamiya *et al*, 1997; Thompson & Steensma, 2006) and a significant fraction of patients developed PRCA after thymectomy (Suzuki *et al*, 2003; Thompson & Steensma, 2006; Hirokawa *et al*, 2008). Although the pathogenesis of thymoma-associated PRCA remains to be elucidated, there exist two potential mechanisms. Thymoma itself alters the subset and/or the repertoires of T lymphocytes, leading to the production of autoaggressive T-cell clones (Hoffacker *et al*, 2000). Another intriguing possibility is that thymectomy may represent a risk for the development of systemic autoimmune disorders over years (Gerli *et al*, 1999).

Thompson and Steensma (2006) reported that surgical resection of thymoma was insufficient for normalization of erythropoiesis in all 13 patients so treated, but immunosuppressive therapy was effective as an adjuvant treatment. Immunosuppressive therapy including CS, CY and CsA has been reported to be useful in cases with thymoma-associated PRCA (Marmont *et al*, 1975; Garcia Vela *et al*, 1993; Charles *et al*, 1996; Thompson & Steensma, 2006), but optimal management of this disorder has remained unclear. Recently, it has been reported that thymoma-associated PRCA showed an excellent response to CsA and CsA-containing regiments were effective in preventing relapse (Hirokawa *et al*, 2008).

Splenectomy, plasmapheresis, and bone marrow transplantation have been used on rare occasions and can be tried if all else fails.

### Peptide-based EPO receptor agonist

Krantz and Kao (1967), for the first time, reported that plasma from a patient with PRCA inhibited haem synthesis by the patient's own bone marrow cells *in vitro*. The serum of patients with anti- EPO antibody-related PRCA also inhibited the growth of erythroid progenitor cells *in vitro* (Casadevall *et al*, 2002). PRCA due to autoantibodies against endogenous EPO occurs but is rare in patients who have never been treated with recombinant human EPO (rhEPO). rhEPO-related PRCA reached a peak incidence mainly in Europe in 2001 to 2002, largely related to a change of formulation, and of uncoated rubber stoppers (leachates present), in a particular rhEPO product, Eprex, and subcutaneous administration (Casadevall *et al*, 2002; Rossert *et al*, 2004).

The two most important initial steps in the management of anti-EPO antibody-mediated PRCA are transfusions for symptomatic anaemia and stopping the administration of rhEPO (Rossert *et al*, 2004). Since PRCA in this setting is immune-mediated, and since spontaneous remissions after cessation of rhEPO therapy are rare, immunosuppressive therapy should be provided in most cases (Verhelst *et al*, 2004; Rossert *et al*, 2005). Anti-rhEPO antibodies cross-react not only with the endogenous hormone, but also with all rhEPO molecules, including darbepoetin alfa (Casadevall *et al*, 2002). Rechallenge with rhEPO preparations may cause an anamnestic antibody response, making it less possible for the antibody to either spontaneously disappear or return to clinically unimportant levels, and may induce the formation of allergic skin and systemic reactions (Weber *et al*, 2002).

Recently, a novel peptide-based EPO receptor agonist called Hematide, which does not cross-react with anti-EPO antibodies, has been developed (Stead *et al*, 2006). Hematide is a synthetic, dimeric peptidic erythropoiesis-stimulating agent covalently linked to polyethylene glycol and is being developed for the treatment of anaemia associated with chronic renal failure and cancer. Because its primary amino acid sequence is unrelated to that of rhEPO, Hematide is unlikely to induce a cross-reactive immune response against endogenous EPO and is reported to correct anaemia induced by anti-rhEPO antibodies in a rat PRCA model (Woodburn *et al*, 2007). Thus, a recent animal study suggested that a possible alternative strategy might be to administer Hematide to patients with PRCA due to anti-rhEPO antibodies, which should enable ongoing stimulation of erythropoiesis.

### Future prospects: proposal for a first-line therapy in primary acquired PRCA

There are several options for inducing remission of PRCA, but many patients with acquired PRCA require immunosuppressive therapy to maintain remissions. As summarized in Table I, CS, CsA and CY plus CS are almost equally effective for inducing remissions of PRCA, but the most important difference between these agents is the feasibility of long-term maintenance. Although the relapsed patients can be re-treated with the same agents, such as CS or CS plus CY, the cumulative side effects and toxicity become unacceptable. Considering the recurrent nature of acquired PRCA, we suggest CsA as first-line therapy for these patients at a dose of 2·5–3 mg/kg twice daily to achieve trough CsA levels of 150–250 ng/ml for a maximum of three to four months. This trough CsA level has been empirically determined according to a consensus from a multicentre randomized study in Japan for aplastic anaemia (Teramura *et al*, 2007). Maintenance therapy with CsA is a requisite for most patients to prevent relapse. Since nephrotoxicity constitutes the major limiting side effect of CsA, careful and progressive decrease of the dosage to the minimum required for maintenance of remission is appropriate. The mean maintenance dose of CsA in Japanese patients who were continuing their first remission for more than 24 months was 2·2 ± 0·8 mg/kg per day with a range of 1·1–3·8 mg/kg per day, 40% of the initial dose (Sawada *et al*, 2007), which suggests difficulty in reducing CsA under this dosage to maintain remissions. Adequate prevention and treatment of infections secondary to immunosuppression are also necessary for successful management of these patients.

**Table I:** Treatment of pure red cell aplasia (PRCA).

Agent	Response rate (CR + PR)*	Mean time to response	Need for maintenance therapy	Feasibility of long-term maintenance§
Corticosteroids; CS (methyl-prednisolone/prednisone/prednisolone)	30–62%	2·5 weeks†	Required† (Most patients relapsed during the taper of CS)	Unacceptable for the dose to maintain remission
		9 weeks in patients with primary idiopathic PRCA‡ (33% of patients achieved remission within 2 weeks)	Required in patients with primary idiopathic PRCA‡	
Cyclosporine A; CsA	65–87%	12 weeks in patients with primary idiopathic PRCA‡ (65% of responders achieved remission within 2 weeks)	Required in patients with primary idiopathic PRCA‡ (86% relapsed after discontinuation of CsA while 11% relapsed during the maintenance of CsA)	May be durable but needs careful monitoring
Cyclophosphamide; CY (CY + CS)	7–20% (40–60%)	11 weeks†	Unknown: required in some patients	Unacceptable

Agent	Relapse free survival (RFS)	Median overall survival (OS)
Corticosteroids; CS (methyl-prednisolone/prednisone/prednisolone)	80% of patients relapse within 24 months after remission during dose-reduction†	14-year OS in patients with primary idiopathic PRCA treated with CS or with various combinations except for CsA†
	Median RFS: 33 months in patients with primary idiopathic PRCA‡ (88% of patients relapsed during CS maintenance)	
Cyclosporine A; CsA	Median RFS: 103 months in patients with primary idiopathic PRCA‡ (Including patients who relapsed after the discontinuation of CsA)	12-year OS in patients with primary idiopathic PRCA responding to remission induction therapy with CsA‡
		10-year OS was 95% and the median OS has not yet been reached in all patients‡
Cyclophosphamide; CY (CY + CS)	Unknown: the duration of remission induced by CY seems to be prolonged as compared to patients induced by CS*	Unknown

Patients with primary acquired and secondary PRCA are included if not otherwise indicated.

* References are indicated in the text.

† Referenced by Clark *et al* (1984).

‡ Referenced by Sawada *et al* (2007).

§ Referenced by Clark *et al* (1984), Sawada *et al* (2007), Radis *et al* (1995) and Reinhold-Keller *et al* (2000).
